# Application of Magnonic Crystals in Magnetic Bead Detection

**DOI:** 10.3390/nano12193278

**Published:** 2022-09-21

**Authors:** Alessandra Manzin, Riccardo Ferrero, Marta Vicentini

**Affiliations:** 1 Istituto Nazionale di Ricerca Metrologica (INRIM), 10135 Torino, Italy; 2 Politecnico di Torino, 10129 Torino, Italy

**Keywords:** magnetic nanostructured films, magnonic crystals, magnetic antidot arrays, magnetic bead detection, magnetic bead trapping, magnetic field sensors, magnetic immunoassays, ferromagnetic resonance, micromagnetic modelling, GPU computing

## Abstract

This paper aims at studying a sensor concept for possible integration in magnetic field-based lab-on-chip devices that exploit ferromagnetic resonance (FMR) phenomena in magnonic crystals. The focus is on 2D magnetic antidot arrays, i.e., magnetic thin films with periodic non-magnetic inclusions (holes), recently proposed as magnetic field sensor elements operating in the gigahertz (GHz) range. The sensing mechanism is here demonstrated for magnetic nano/microbeads adsorbed on the surface of permalloy (Ni_80_Fe_20_) antidot arrays with a rhomboid lattice structure and variable hole size. Through extensive micromagnetic modelling analysis, it is shown that the antidot arrays can be used as both bead traps and high-sensitivity detectors, with performance that can be tuned as a function of bead size and magnetic moment. A key parameter for the detection mechanism is the antidot array hole size, which affects the FMR frequency shifts associated with the interaction between the magnetization configuration in the nanostructured film and the bead stray field. Possible applications of the proposed device concept include magnetic immunoassays, using magnetic nano/microbeads as probes for biomarker detection, and biomaterial manipulation.

## 1. Introduction

In the last two decades, advances in nanostructure fabrication have driven the development of miniaturized magnetic sensors for innovative applications in the industrial, automotive, aerospace, ICT, metrology and calibration, green technology, and medical fields [1,2,3,4,5,6,7,8,9,10,11]. In biomedicine, they can be employed in the detection of functionalized magnetic labels—e.g., magnetic nanoparticles or nano/microbeads—for molecular sensing and targeting, drug delivery, sample purification, and cell manipulation [5,6,7,8,9,10,11]. In the panorama of magnetic biosensors, magnetoresistance- and Hall-effect-based devices have optimal properties, offering very high sensitivity and magnetic field resolution, as well as easy miniaturization and lab-on-chip integration [5,6,7,8,9,10,11,12,13,14,15].

Another way to measure low magnetic fields, such as those produced by magnetic nanoparticles or nano/microbeads, is to exploit the ferromagnetic resonance (FMR) modes of spintronic devices [16,17], magnonic crystals [18,19,20,21], magnetic disks [22], nanoparticles [23,24], or thin films [25]. This technology has the advantage of reducing the impact of low-frequency 1/*f* noise [16]. Recently, it was shown that magnonic crystals based on magnetic nanostructured films can be employed as high-sensitivity magnetic field detectors, exploiting their dynamic behavior in the gigahertz (GHz) range [19,20,21]. The sensing mechanism was demonstrated for the case of magnetic nano/microparticles trapped on the surface of 2D permalloy (Ni_80_Fe_20_) antidot arrays, i.e., magnetic thin films nanostructured with periodic closely packed non-magnetic inclusions (holes) [26]. In particular, the magnetic stray field generated by the particles interacts with the magnetization state of the antidot array, producing a measurable shift in its FMR frequencies or FMR fields [19,20,21]. The geometric properties of the nanostructured film lattice can be tuned to maximize the generated signal, considering that both the static and dynamic behaviors of magnetic antidot arrays are strongly influenced by the lattice geometry and by the hole size, density, and shape [26,27,28,29,30,31,32].

In this study, we investigated with micromagnetic modelling the dynamic response of 2D permalloy antidot arrays with a rhomboid lattice structure and circular holes, under the influence of the stray field generated by magnetic beads dispersed on the nanostructured film. The simulations were performed by assuming the beads to be immobilized on the film surface in correspondence of the holes, where the magnetic interaction should be stronger. This hypothesis was corroborated by a preliminary evaluation of the magnetic force (attractive or repulsive) exerted by the antidot array on the magnetic beads, for different values of the applied bias field.

We performed an extensive parametric analysis, varying the bead size and saturation magnetic moment as well as the diameter of the antidot array holes. The final objective was to find the optimal geometrical configuration of the nanostructured film in terms of magnetic particle trapping and magnetic field sensitivity, as a function of the properties of the magnetic beads to be manipulated and detected. Our attention was focused on the shifts in the FMR frequencies of the antidot array, caused by the presence of the beads. The evaluation of the FMR behavior was performed by means of a parallelized micromagnetic code, which was engineered to run on graphics processing units (GPUs) to efficiently solve the Landau–Lifshitz–Gilbert (LLG) equation in large magnetic objects [33].

## 2. Materials and Methods

### 2.1. Micromagnetic Simulations

The micromagnetic simulations were performed by means of an in-house numerical code, which was implemented to solve the LLG equation by exploiting parallel computing on GPUs [33]. The input geometry was a grid of macrocells, representing the spatial period of the magnetic antidot array, and discretized into *T* hexahedral elements with size in the range of the permalloy exchange length (~5 nm) [34]. The magnetization vector **M**, assumed to be uniform in each hexahedron (centered on **r**_0_), was updated by time-integrating the LLG equation:

(1) $$\frac{\partial M\left(r_{0}\right)}{\partial t}=-\frac{\gamma }{1+\alpha^{2}}M\left(r_{0}\right)\times {\left\{H_{\mathrm{eff}}\left(r_{0}\right)+\frac{\alpha }{M_{S}}\left[M\left(r_{0}\right)\times H_{\mathrm{eff}}\left(r_{0}\right)\right]\right\}}_{,}$$

where *M*_S_ is the saturation magnetization, γ = 2.21 · 10^5^ m A^−1^ s^−1^ is the absolute value of the gyromagnetic ratio, and α is the damping coefficient. In Equation (1), the effective field **H**_eff_ is the sum of the applied field **H**_a_ (spatially uniform for the studied cases), the magnetostatic field **H**_m_, the exchange field **H**_ex_, and the magnetocrystalline anisotropy field **H**_an_ (negligible for permalloy). **H**_eff_ can also include the stray field produced by magnetic beads, namely, **H**_beads_.

The exchange field **H**_ex_ was calculated with a non-standard finite difference technique able to handle non-structured meshes, as detailed in [35].

The demagnetizing field **H**_m_ was computed with a fast multipole technique, after being decomposed into a short- and a long-range term [36,37]. The short-range term (**H**_m_^SR^) includes the dipole–dipole interactions between the hexahedron of calculus (centered on **r**_0_) and the hexahedra belonging to *N*_near_ macrocells, which comprise the one containing **r**_0_ and the adjacent ones. In particular, **H**_m_^SR^ was calculated by integrating and summing the Green’s function gradients over the boundary (∂Ω*_i_*) of each hexahedron (with the unit normal vector **n***_i_*) within the *N*_near_ macrocells:

(2) $$H_{m}^{\mathrm{SR}}\left(r_{0}\right)=\frac{1}{4\pi }{\sum_{n=1}^{N_{\mathrm{near}}}\sum_{i=1}^{T}M\left(r_{i}\right)\cdot n_{i}\int_{\partial \Omega_{i}}\frac{\left(r_{0}-r_{i}\right)}{{\left\|r_{0}-r_{i}\right\|}^{3}}ds_{i}}_{,}$$

where *N*_near_ = (2λ+1)^2^, and λ is the number of layers of the adjacent macrocells considered in the approximation.

The long-range term (**H**_m_^LR^) includes the dipole–dipole interactions with the remaining *N*_far_ macrocells and was calculated via the multipole expansion technique with order *p*:

(3) $$H_{m}^{\mathrm{LR}}\left(r_{0}\right)=-\frac{1}{4\pi }\sum_{f=1}^{N_{\mathrm{far}}}\sum_{j=0}^{p}\;\sum_{k=-j}^{j}A_{f,j}^{k}\left(O_{0}\right)\nabla R_{j}^{k}\left(r_{0}-O_{0}\right),$$

where the time-dependent function $A_{f,j}^{k}\left(O_{0}\right)$ incorporates the contributions to the macrocell of calculus (centered on **O**_0_) of all the multipole moments associated with the *N*_far_ macrocells. The term $\nabla R_{j}^{k}$ depends on the spatial discretization only, and was evaluated at the beginning of the simulation.

**H**_m_ was calculated with the lowest multipole expansion order (*p* = 2) and λ = 1; this choice of parameters enables us to strongly reduce the number of stored terms and the computational complexity, while also maintaining a good level of accuracy [34].

The LLG equation was time-integrated by means of a norm-conserving scheme based on the Cayley transform and the Heun algorithm [38,39]. As discussed in [40], this approach guarantees an efficient determination of equilibrium points, such as the ground states for the dynamic response.

### 2.2. Calculation of Bead Stray Field and Magnetic Force

In Equation (1), the stray field produced by the magnetic beads was calculated as follows:

(4) $$H_{\mathrm{beads}}\left(r_{0}\right)=\frac{1}{4\pi }\sum_{n=1}^{N_{\mathrm{beads}}}\left\{\frac{3\left(r_{n}-r_{0}\right)\left[m_{\mathrm{bead},n}\cdot \left(r_{n}-r_{0}\right)\right]}{{\left\|r_{n}-r_{0}\right\|}^{5}}-\frac{m_{\mathrm{bead},n}}{{\left\|r_{n}-r_{0}\right\|}^{3}}\right\},$$

where *N*_beads_ is the number of beads, **m**_bead,*n*_ is the magnetic moment of the *n*-th bead, and **r***_n_* is the vector position of its barycenter [41]. The bead magnetic moment was approximated by means of the Langevin function:

(5) $$m_{\mathrm{bead},n}=m_{S}\left\{\coth\left[\frac{H_{a}+H_{\mathrm{film}}\left(r_{n}\right)}{H_{0}}\right]-\left[\frac{H_{0}}{H_{a}+H_{\mathrm{film}}\left(r_{n}\right)}\right]\right\},$$

where *m*_S_ and *H*_0_ are the bead saturation magnetic moment and characteristic field, respectively, assumed to be the same for all the beads. These two parameters were derived by fitting the experimental magnetization curve of the beads. **H**_film_ is the stray field produced by the nanostructured magnetic film (i.e., the antidot array) in correspondence of the bead barycenter **r***_n_*, calculated once known the magnetization vector in all the *Q* hexahedra discretizing the entire film. **H**_film_ was determined as follows:

(6) $$H_{\mathrm{film}}\left(r_{n}\right)=\frac{1}{4\pi }{\sum_{i=1}^{Q}M\left(r_{i}\right)\cdot n_{i}\int_{\partial \Omega_{i}}\frac{\left(r_{n}-r_{i}\right)}{{\left\|r_{n}-r_{i}\right\|}^{3}}ds_{i}}_{.}$$

The magnetic force that the magnetic antidot array exerts on the *n*-th bead is expressed as follows:

(7) $$F_{\mathrm{mag}}\left(r_{n}\right)=\mu_{\mathrm{fluid}}\left(m_{\mathrm{bead},n}\cdot \nabla \right)H_{\mathrm{film}}{\left(r_{n}\right)}_{,}$$

where μ_fluid_ is the magnetic permeability of the fluid in which the beads are immersed, here assumed to be equal to the vacuum permeability μ_0_ [41,42].

### 2.3. Micromagnetic Simulation Parameters

The micromagnetic simulations were performed by considering magnetic parameters typical of permalloy, i.e., the magnetization saturation was set to 860 kA/m, the exchange constant was set to 13 pJ/m, and the magnetocrystalline anisotropy was neglected. Regarding the choice of the damping coefficient α for the modelling of the static and dynamic regimes, we adopted the approach proposed in [43]. In particular, the FMR behavior was determined by fixing α to a realistic value for non-doped (pure) permalloy, i.e., α = 0.01 [44]; the corresponding time evolution simulations were carried out with a time step of 250 fs. The ground states for the dynamic response were computed with α = 0.1; the increase in α was motivated by the need to reduce the computational time in the determination of the magnetization equilibrium configurations, as described in [40]. With such a value of the damping coefficient, the convergence to equilibrium can be indeed accelerated and, with the Cayley-transform-based time-integration scheme, time steps in the order of 2.5 ps can be used without causing numerical instability [38].

## 3. Results and Discussion

**Figure 1:**
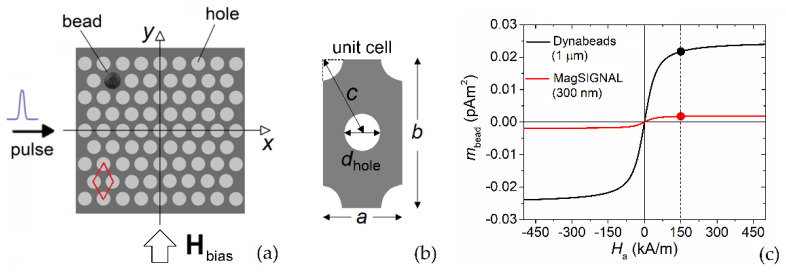
(**a**) Schematic of the permalloy nanostructured film (antidot array with rhomboid lattice structure). The schematic includes the configuration of the bias field and of the FMR excitation with a Gaussian pulse waveform. (**b**) Schematic of the unit cell of the antidot array, with relevant geometric parameters. (**c**) Magnetization curves of MagSIGNAL-STA and Dynabeads MyOne beads, fitted to the experimental data from the manufacturers. The dots indicate the magnetic moment values when a bias field of 150 kA/m is applied.

Our analysis was focused on permalloy antidot arrays with a rhomboid lattice structure [26] and circular holes with diameter *d*_hole_ (variable from 250 nm to 400 nm) and center-to-center hole distance *c* fixed to 500 nm. The considered nanostructured film, schematized in Figure 1a together with the configuration of the FMR excitation, has a thickness of 10 nm and a side length of 4.5 µm. The lattice with the unit cell depicted in Figure 1b is characterized by easy and hard axes whose directions depend on the geometric parameters *d*_hole_ and *c*. As described in [27], for *d*_hole_ = 250 nm the easy axes are aligned along the directions where the non-magnetic inclusions are closest to one another. These correspond to the *x*-axis and the geometrically equivalent directions, shifted in multiples of 60°, while the hard axes are parallel to the directions where the non-magnetic inclusions are farthest, such as the *y*-axis. A different anisotropy behavior was found when *d*_hole_ = 400 nm, as detailed in [27].

Two types of commercial magnetic particles were considered for the detection, namely, MagSIGNAL-STA beads from MagnaMedics GmbH (Aachen, Germany), and Dynabeads MyOne beads from Thermo Fisher Scientific (Carlsbad, CA, USA), developed for use as detection labels in magnetic immunoassays [45]. The former have a diameter *d*_bead_ of 300 nm and a saturation magnetic moment around 0.002 pAm^2^, while the latter have a diameter of 1 µm and a saturation magnetic moment around 0.0245 pAm^2^; the fitting magnetization curves, determined using Equation (5), are reported in Figure 1c. To prove that the beads could be trapped at the antidot array holes, we first calculated the spatial distribution of the magnetic force exerted on the beads by the nanostructured film. Then, we simulated the FMR behavior of the film, comparing the FMR spectra obtained with and without beads immobilized on the film surface.

### 3.1. Analysis of Bead Trapping

**Figure 2:**
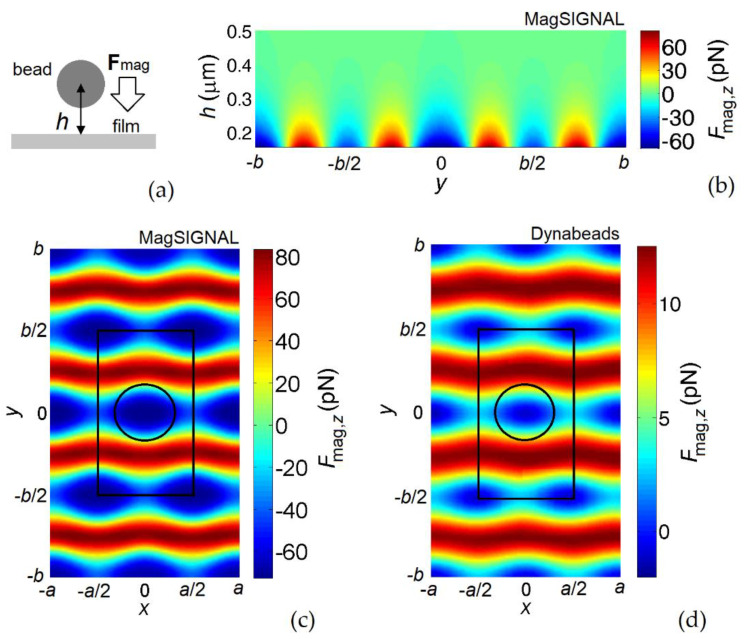
(**a**) Schematic of bead trapping. (**b**) Map of the vertical component of the magnetic force for MagSIGNAL-STA beads, calculated on a plane orthogonal to the permalloy nanostructured film (with *d*_hole_ = 330 nm) and parallel to the longer side of the unit cell. Maps of the vertical component of the magnetic force calculated on a plane parallel to the film for (**c**) MagSIGNAL-STA and (**d**) Dynabeads MyOne beads. The beads are assumed to be in contact with the film surface. For all maps, the applied bias field is equal to 150 kA/m.

The mechanism of bead trapping was investigated by calculating the magnetic force **F**_mag_ due to the stray field of the permalloy antidot array **H**_film_, as a function of bead height *h*, i.e., the vertical distance between the bead barycenter and the film top surface (Figure 2a). The hole diameter was fixed to 330 nm. First, we assumed that the film was quasi-saturated along the *y*-axis with an applied bias field with amplitude equal to 150 kA/m. Figure 2b shows the map of the vertical component of **F**_mag_ estimated for the MagSIGNAL-STA beads on a plane orthogonal to the film and parallel to the longer side of the unit cell (*yz*-plane). The force becomes negligible when *h* is greater than 0.5 µm; in proximity to the film surface, the periodic spatial distribution of the force vertical component is well evident, with negative peaks over the holes (attractive action) and positive peaks over the striped regions along the *x*-axis not containing holes (repulsive action). The alternation of positive and negative forces is well evident in Figure 2c, which depicts the spatial distribution of the vertical component of **F**_mag_ estimated for the MagSIGNAL-STA beads on a plane parallel to the film (*xy*-plane), when the beads are in contact with the film surface (*h* = *d*_bead_/2). The maximum attractive force is 72 pN, while the maximum repulsive force is 83 pN; lower attractive forces (around 45 pN) can be found between the holes. The magnetostatic coupling is weaker for the 1 µm beads (Dynabeads MyOne), with peaks of the attractive force in the order of 2 pN at the lowest bead–film distance (Figure 2d).

**Figure 3:**
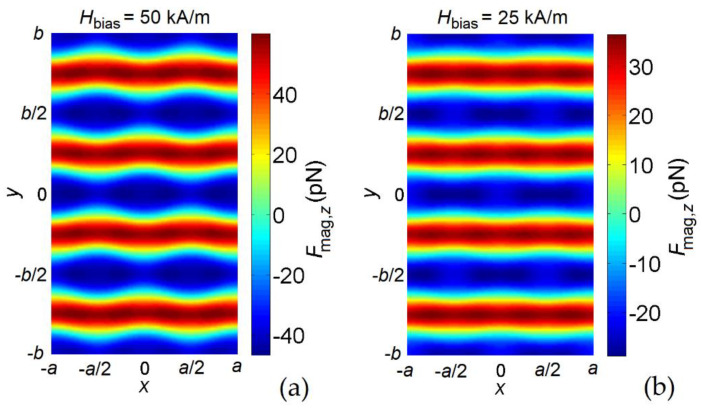
Maps of the vertical component of the magnetic force calculated on a plane parallel to the permalloy nanostructured film for MagSIGNAL-STA beads, applying a bias field of (**a**) 50 kA/m and (**b**) 25 kA/m. The beads are assumed to be in contact with the film surface.

The decrease in the applied bias field leads to a gradual reduction in **F**_mag_ and to a strong modification of its spatial distribution, as demonstrated by Figure 3, which presents the results obtained for the MagSIGNAL-STA beads when *H*_bias_ is equal to 50 kA/m (Figure 3a) and 25 kA/m (Figure 3b). Because of the change in the film demagnetizing field pattern driven by the reduction in magnetostatic energy, the stray field lines are strongly modified, and for the lowest bias field value the peaks of the attractive force no longer appear over the holes, but over the regions between the holes. In correspondence of the hole centers, for the MagSIGNAL-STA beads the vertical component of **F**_mag_ varies from −44 pN to −23 pN when reducing *H*_bias_ from 50 kA/m to 25 kA/m, with an overall decrease of 68% from *H*_bias_ = 150 kA/m. Over the regions between the holes the variation ranges from −40 pN to −28 pN, with an overall decrease of 38% from *H*_bias_ = 150 kA/m.

The obtained results prove that magnetic antidot arrays can be used as traps for magnetic beads, with a local variation of the magnetic force depending on the lattice geometry and the applied magnetic field amplitude, as well as on the bead properties (i.e., size and magnetic moment). It can be observed that the holes act as preferential adsorption sites for magnetic beads when strong external fields are applied. In this case, the film is quasi-saturated, leading to strong stray fields able to strongly interact with the beads when they are in proximity to the film surface after gravimetric deposition. This interaction is greater for the smaller beads, due to the possibility of closely approaching the nanostructured film surface, where large stray field gradients are present.

### 3.2. Analysis of FMR-Based Bead Detection

The mechanism of magnetic bead detection was investigated under the assumption that the beads were immobilized on the film surface in correspondence of the holes, where the magnetic interaction is expected to be stronger for sufficiently large bias fields (see Section 3.1). The operation of the magnetic antidot array as an FMR-based sensor was studied by applying a quasi-saturating direct current (DC) bias field of 150 kA/m along the *y*-axis and an excitation field along the *x*-axis with the following Gaussian pulse waveform:

(8) $$H_{\mathrm{pulse}}\left(t\right)=a\exp{\left[-{\left(\frac{t-t_{0}}{\tau }\right)}^{2}\right]}_{,}$$

with an amplitude *a* of 1 kA/m and an RMS width τ of 5 ps. Both the DC and excitation fields are spatially uniform.

Due to the large bias field, the bead–bead magnetostatic interactions can be disregarded. Indeed, they are practically negligible, leading to a maximum variation in the bead magnetic moment of 2% for the cases when the interaction is stronger (i.e., in the presence of bead stratification over the film surface). This variation was previously estimated using the iterative approach described in [14].

#### 3.2.1. Influence of Bead Type

**Figure 4:**
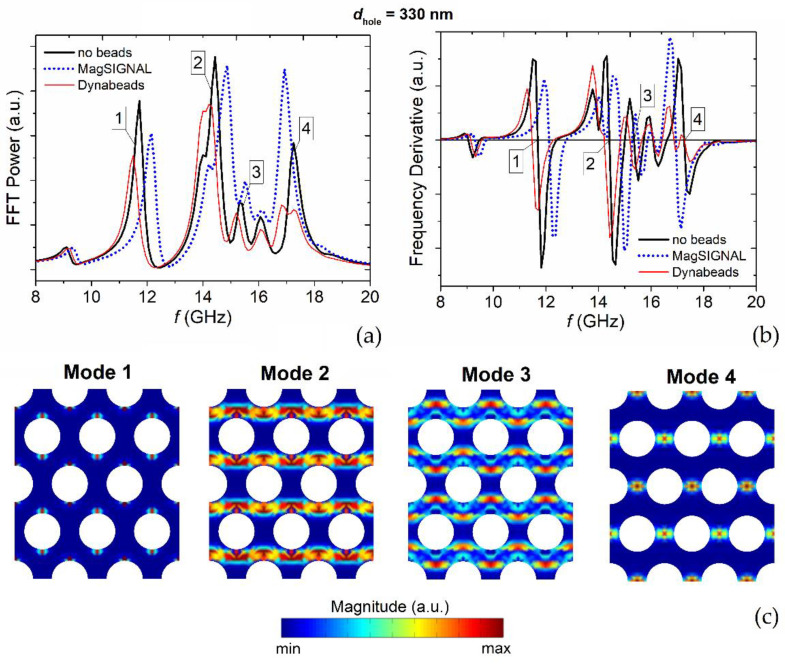
(**a**) FFT power spectra of the average magnetization component along the excitation field direction (*x*-axis), for a bias field of 150 kA/m parallel to the *y*-axis. The graph shows the FMR responses of the permalloy nanostructured film with 330 nm holes to the stray fields generated by the MagSIGNAL-STA and Dynabeads MyOne beads trapped at the holes, assuming that the film surface is fully covered by beads. The case without beads is also reported for comparison. (**b**) Corresponding frequency derivatives of the FFT power spectra. (**c**) Surface plots of Fourier coefficient magnitude for the FMR modes indicated in (**a**) and (**b**), calculated in a magnified area of the film for the case without beads.

Figure 4a reports the FFT power spectra (in arbitrary units) of the spatially averaged magnetization component along the excitation field direction (*x*-axis); the corresponding frequency derivatives are shown in Figure 4b. The calculations were performed assuming the film surface to be fully covered by beads (trapped at the holes). This means that for the MagSIGNAL-STA beads, all the antidot array holes are filled by beads, while for the Dynabeads MyOne beads only 30% of the holes are filled. The results were obtained by fixing the hole diameter to 330 nm. The graph compares the curves calculated with both MagSIGNAL-STA and Dynabeads MyOne beads to the result obtained in the absence of beads (reference case). The reference FFT spectrum displays four well-defined peaks around 11.7 GHz, 14.4 GHz, 15.4 GHz, and 17.3 GHz. The localization of the resonant modes in the antidot array can be inferred by analyzing the spatial distributions of the corresponding spin precession amplitudes, as shown in Figure 4c for a magnified area of the sample. The FMR modes are distinguished following the well-known classification in edge, extended, and localized modes [46], considering that the FMR frequencies depend on the effective field in the sample, which is practically the sum of the bias and demagnetizing fields for the considered case [47,48]. The exchange field is indeed negligible, with the film being magnetically saturated.

**Figure 5:**
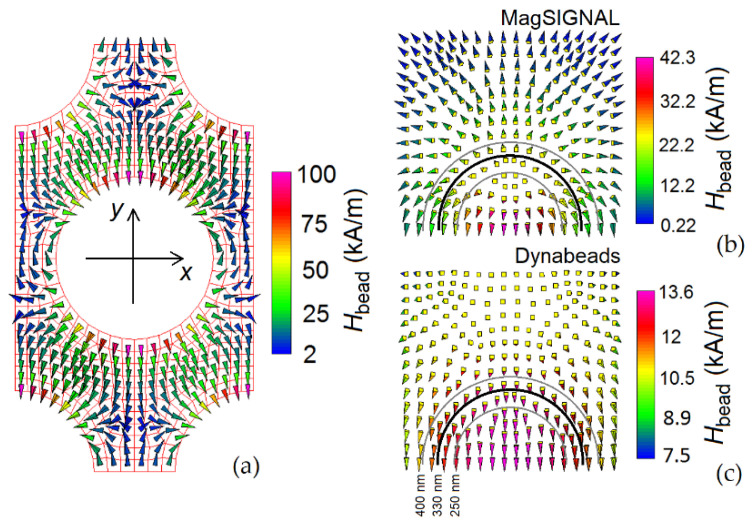
(**a**) Spatial distribution of the demagnetizing field in the unit cell of the permalloy nanostructured film with 330 nm holes. Spatial distribution of the stray field produced on the film surface by (**b**) MagSIGNAL-STA and (**c**) Dynabeads MyOne beads, centered with the film holes and in contact with the film surface.

As demonstrated by the relative spin-wave profile, the lowest-frequency mode is an edge mode (mode #1). Indeed, the highest spin precession amplitude associated with this mode is found at the hole edges crossed by the *y*-axis, where the demagnetizing field reaches peak values opposite to the bias field [49,50,51,52] (see the spatial distribution of the demagnetizing field calculated within the antidot array unit cell, reported in Figure 5a). The modes at intermediate frequencies are extended or quasi-uniform modes distributed throughout the whole lattice along stripes orthogonal to the bias field, where the demagnetizing field decreases from ∼25 kA/m (mode #2) down to zero (mode #3). The final mode is a localized one (mode #4), with the highest spin precession amplitude localized between neighboring holes, where the demagnetizing field changes sign.

As shown in Figure 4a and Table 1, the presence of MagSIGNAL-STA beads produces a clear shift in the FMR frequencies of all the modes, equal to 0.43 GHz, 0.40 GHz, 0.17 GHz, and −0.31 GHz for the edge, first extended, second extended, and localized modes, respectively. Conversely, the Dynabeads MyOne beads are responsible for a shift of −0.22 GHz, −0.19 GHz, −0.16 GHz, and −0.38 GHz for the edge, first extended, second extended, and localized modes, respectively.

The sign of the frequency shifts in the FMR modes depends on the bead type. This can be understood by analyzing the spatial distribution of the antidot array demagnetizing field (Figure 5a) and of the stray field generated by a hole-centered magnetic bead at the film surface. It is important to consider that the FMR frequency increases if the stray field of the bead, when interacting with the magnetization spatial distribution in the antidot array, leads to an increase in the effective field in the antidot array itself. For a bias field of 150 kA/m, the effective field is indeed dominated by the applied field contribution, directed along the positive *y*-axis. For the MagSIGNAL-STA bead (Figure 5b), having a size comparable to that of the holes, the bead stray field opposes the film demagnetizing field at the hole edges crossed by the *y*-axis, leading to an increase in the FMR frequency of the edge mode. This also occurs for the regions in which the first extended mode is prevalent. In the areas dominated by the second extended mode, the bead produces a non-negligible stray field with the same sign as the bias field, leading to a positive shift in the associated FMR frequency. Finally, in the regions between neighboring holes, where the localized mode appears, the bead stray field opposes both the bias and demagnetizing fields, with a consequent reduction in the corresponding FMR frequency. For the Dynabeads MyOne bead (Figure 5c), the stray field opposes the bias field for all modes, leading to a decrease in the FMR frequencies for all modes.

#### 3.2.2. Influence of Hole Size

**Figure 6:**
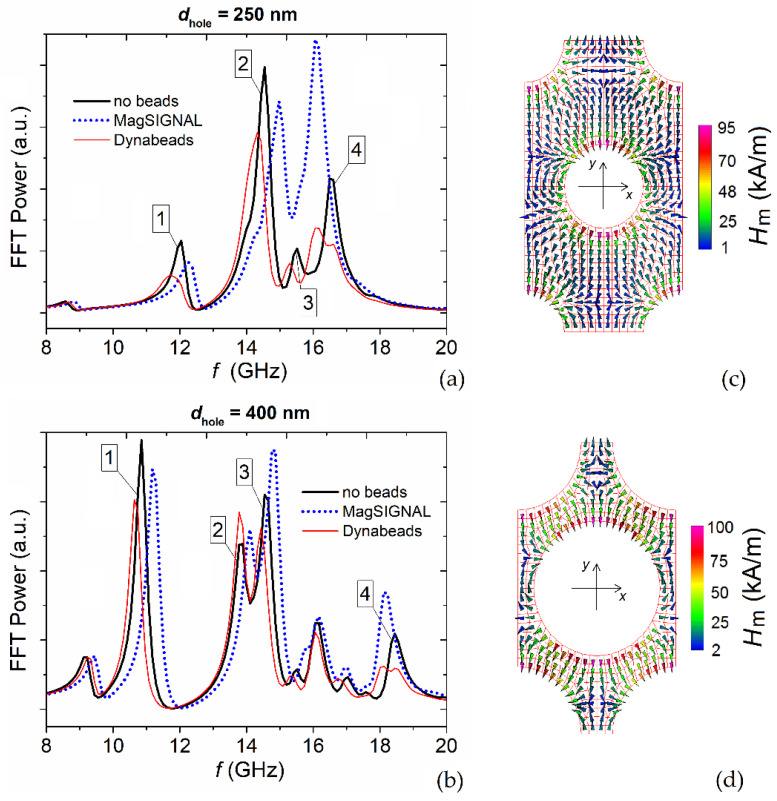
FFT power spectra of the average magnetization component along the excitation field direction (*x*-axis) for a bias field of 150 kA/m parallel to the *y*-axis. The graph shows the FMR responses of the permalloy nanostructured film with (**a**) 250 nm and (**b**) 400 nm holes to the stray fields generated by MagSIGNAL-STA and Dynabeads MyOne beads trapped in the holes, assuming that the film surface is fully covered by beads. The case without beads is also reported for comparison. The numbers refer to the main modes in the spectra. Spatial distributions of the demagnetizing field in the unit cell of the permalloy nanostructured film with (**c**) 250 nm and (**d**) 400 nm holes.

The influence of the antidot array hole size on magnetic bead detection was investigated by calculating the FMR spectra for *d*_hole_ equal to 250 nm (Figure 6a) and 400 nm (Figure 6b), in the presence of both MagSIGNAL-STA and Dynabeads MyOne beads fully covering the surface of the permalloy nanostructured film. The shifts in the FMR frequencies of the main modes, caused by the bead stray field, are reported in Table 1. For a better understanding of the influence of *d*_hole_ on the FMR frequency shifts, the spatial distributions of the demagnetizing field calculated within the antidot array unit cell are reported in Figure 6c,d for *d*_hole_ equal to 250 nm and 400 nm, respectively.

When *d*_hole_ = 250 nm, the FMR spectra are characterized by a reduced range [31] and by only three modes, i.e., the edge (mode #1), the first extended (mode #2), and the localized mode (mode #4), tending towards the FMR spectrum of the non-structured (continuous) film, which presents one mode. When *d*_hole_ = 400 nm, the FMR spectra are more extended, with the appearance of additional modes. Focusing on the edge mode (mode #1), the increase in *d*_hole_ leads to a larger positive shift in the relative frequency for the MagSIGNAL-STA beads, because of a more pronounced anti-parallelism of the bead stray field (Figure 5b) and the demagnetizing field (Figure 6d) at the hole edges crossed by the *y*-axis. For the Dynabeads MyOne beads, the amplitude of the negative shift decreases due to a reduction in the component of the bead stray field (Figure 5c) parallel to the demagnetizing field (Figure 6d). For the extended modes (modes #2 and #3, if present), the amplitudes of the FMR frequency shifts decrease with *d*_hole_ for both bead types. This is associated with shrinkage in the nanostructured film areas where these modes appear. Finally, the increase in *d*_hole_ leads to a limited decrease in the FMR frequency of the localized mode (mode #4) for both beads, due to a reduction in the amplitude of the bead stray field (opposite to the demagnetizing field) in the areas of interest.

In summary, for the MagSIGNAL-STA beads, the increase in *d*_hole_ from 250 nm to 400 nm enhances the detection mechanism that exploits variations in the edge mode frequency. Conversely, for the other modes, it is convenient to reduce *d*_hole_ to obtain larger shifts in the FMR frequencies. The best performance is achieved with *d*_hole_ = 400 nm for the edge mode, leading to a frequency variation around 1 GHz (Table 1). Variations of 0.45 GHz are obtained for the first extended and localized modes when *d*_hole_ = 250 nm. Worse performance is found in the case of Dynabeads MyOne bead detection, for which the best sensitivity is obtained with *d*_hole_ = 250 nm when exploiting the localized mode. This is a consequence of the reduced fraction of antidot array holes filled by 1 μm beads, and of a weaker coupling between their stray field and the magnetization within the film, for the range of *d*_hole_ variation considered here.

#### 3.2.3. Influence of Film Coverage

**Figure 7:**
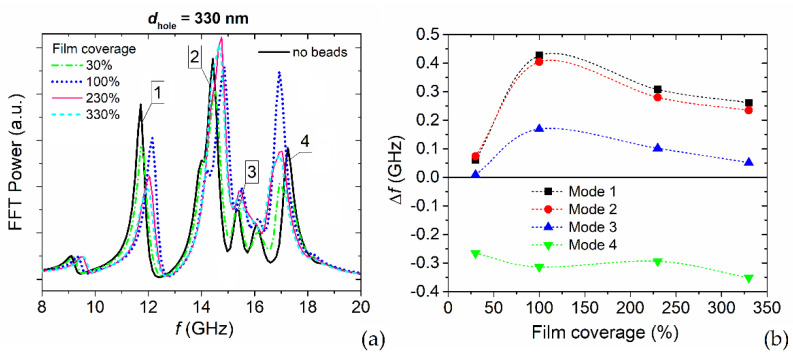
(**a**) FFT power spectra of the average magnetization component along the excitation field direction (*x*-axis) for a bias field of 150 kA/m parallel to the *y*-axis. The graph shows the FMR responses of the permalloy nanostructured film with 330 nm holes to the stray field generated by MagSIGNAL-STa beads, as a function of film coverage. (**b**) Corresponding FMR frequency shifts for the edge (#1), first extended (#2), second extended (#3), and localized (#4) modes, versus film coverage percentage.

Finally, we investigated the influence of bead concentration on the FMR spectra of the permalloy nanostructured film, by varying the percentage of film coverage from 30% to 330%. We focused on MagSIGNAL-STA beads, for which a coverage of 100% means that each hole of the antidot array is filled by one bead, while coverages of 230% and 330% are associated with a two- and three-layer stratification over the film surface, respectively. Figure 7a shows the FMR spectra for a film with *d*_hole_ = 330 nm, calculated again for a bias field of 150 kA/m parallel to the *y*-axis and an excitation field applied along the *x*-axis. Figure 7b reports the relative FMR frequency variations associated with the main modes.

It is worth noting that for the first three modes there is a strong increase in the frequency variations up to 100% film coverage. In the presence of bead stratification, there is a reduction in the signal, caused by a limited anti-parallelism of the demagnetizing field and the bead stray fields in the areas interested by such modes. For larger bead distances with respect to the film surface, the vertical component of the stray field becomes dominant, with a strong decrease in the component along the *y*-axis. For the fourth mode, a less significant change is found, with Δ*f* within the range −0.35–−0.25 GHz, due to a reduced variation of the stray field orientation with the distance.

In summary, the edge and first extended modes are associated with the most significant frequency shifts when the film coverage is 100%, and can be used for preliminary bead quantification in sensor devices.

## 4. Conclusions

The micromagnetic modelling analysis carried out in this study demonstrates that permalloy antidot arrays can be used for trapping, manipulating, and detecting magnetic nano/microbeads, paving the way for their integration into lab-on-chip devices for biomedical applications. In addition to magnetic immunoassays for biomarker quantification, these nanostructured films could be also employed for developing biomaterial manipulators and magnetic scaffolds for tissue engineering, exploiting magnetic bead trapping.

The trapping mechanism was found to be enhanced in the presence of large bias fields, which quasi-saturated the permalloy nanostructured film, guaranteeing high gradients of the film stray field and, thus, large attractive forces, in correspondence of the holes. The interaction was greater for the smaller beads, due to their ability to more closely approach the film surface.

The detection mechanism, which exploits the dynamic behavior in the GHz range and the shifts in the FMR frequencies caused by the interaction between the bead stray field and the antidot array magnetization state, can be maximized by a proper choice of hole size and FMR mode to be monitored. This optimization must be addressed by the properties of the beads to be detected (i.e., size and magnetic moment). For example, FMR frequency variations up to 1 GHz were observed when considering 400 nm holes and exploiting the edge mode for detecting 300 nm beads with a magnetic moment around 0.002 pAm^2^_._

It is worth noting that multilayered stratification of beads over the nanostructured film surface generally leads to a reduction in the detected signal, negatively impacting on accurate bead quantification in a wide range of concentrations. The edge and first extended modes are associated with the greatest frequency shifts when the film coverage is 100%, and can be used for preliminary bead quantification in sensor devices in the presence of moderate bead concentrations that do not lead to multilayered stratification.

## Figures and Tables

**Table 1:** Shifts in the FMR frequencies associated with the edge (#1), first extended (#2), second extended (#3), and localized (#4) modes, as a function of the diameter of the antidot array holes and the types of beads to be detected.

Bead Type	*d*_hole_ (nm)	Δ*f*_1_ (GHz)	Δ*f*_2_ (GHz)	Δ*f*_3_ (GHz)	Δ*f*_4_ (GHz)
MagSIGNAL	250	+0.26	+0.45	−	−0.45
MagSIGNAL	330	+0.43	+0.40	+0.17	−0.31
MagSIGNAL	400	+0.99	+0.26	+0.22	−0.28
Dynabeads	250	−0.27	−0.19	−0.21	−0.43
Dynabeads	330	−0.22	−0.19	−0.16	−0.38
Dynabeads	400	−0.17	−0.03	−0.16	−0.36

## Data Availability

The data presented in this study are available on request from the corresponding author.
